# Organophosphorus pesticides exhibit compound specific effects in rat precision-cut lung slices (PCLS): mechanisms involved in airway response, cytotoxicity, inflammatory activation and antioxidative defense

**DOI:** 10.1007/s00204-021-03186-x

**Published:** 2021-11-15

**Authors:** Jonas Tigges, Franz Worek, Horst Thiermann, Timo Wille

**Affiliations:** 1 Bundeswehr Institute of Pharmacology and Toxicology, Neuherbergstrasse 11, 80937 Munich, Germany; 2 Present Address: Department CBRN Medical Defence, Bundeswehr Medical Academy, Ingolstädter Str. 240, 80939 Munich, Germany

**Keywords:** Organophosphates, PCLS, Inflammation, Oxidative stress, Bronchoconstriction

## Abstract

Organophosphorus compound pesticides (OP) are widely used in pest control and might be misused for terrorist attacks. Although acetylcholinesterase (AChE) inhibition is the predominant toxic mechanism, OP may induce pneumonia and formation of lung edema after poisoning and during clinical treatment as life-threatening complication. To investigate the underlying mechanisms, rat precision-cut lung slices (PCLS) were exposed to the OP parathion, malathion and their biotransformation products paraoxon and malaoxon (100–2000 µmol/L). Airway response, metabolic activity, release of LDH, cytokine expression and oxidative stress response were analyzed. A concentration-dependent inhibition of airway relaxation was observed after exposure with the oxon but not with the thion-OP. In contrast, cytotoxic effects were observed for both forms in higher concentrations. Increased cytokine expression was observed after exposure to parathion and paraoxon (IL-6, GM-CSF, MIP-1α) and IL-6 expression was dependent on NFκB activation. Intracellular GSH levels were significantly reduced by all four tested OP but an increase in GSSG and HO-1 expression was predominantly observed after malaoxon exposure. Pretreatment with the antioxidant *N*-acetylcysteine reduced malaoxon but not paraoxon-induced cytotoxicity. PCLS as a 3D lung model system revealed OP-induced effects depending on the particular OP. The experimental data of this study contribute to a better understanding of OP toxicity on cellular targets and may be a possible explanation for the variety of clinical outcomes induced by different OP.

**Supplementary Information:**

The online version contains supplementary material available at 10.1007/s00204-021-03186-x.

## Introduction

Organophosphorus compound pesticides (OP) are a class of highly toxic substances commonly used for crop protection. The widespread use and easy access resulted in high numbers of (self-) poisonings during the past decades (Peter et al. 2014; Eddleston et al. 2005). A conservative estimate suggests more than 100.000 death per year from pesticide self-poisoning worldwide, accounting for 13.7% of global suicides (Mew et al. 2017).

OP like parathion and malathion require biotransformation by cytochrome P450 enzymes to obtain the more toxic oxon-forms (Fukuto 1990; Eyer et al. 2003). The activated OPs exhibit acute toxicity via covalent binding to the active site of the AChE, leading to its inhibition. The subsequent accumulation of acetylcholine (ACh) at the acetylcholine receptor leads to a cholinergic crisis characterized by a toxidrome of muscarinic and nicotinic signs involving miosis, salivation, bronchospasm, bronchorrhea, muscular dysfunction and respiratory paralysis (Holmstedt 1959; Johnson 1987).

Standard treatment involves application of atropine, to antagonize muscarinic effects, in combination with an oxime for reactivation of inhibited AChE (Worek et al. 2020; Hrabetz et al. 2013). Even if therapeutic steps are rapidly induced and the clinical signs of cholinergic crisis mitigate, OP-induced pneumonia poses a life-threatening complication (Kamat et al. 1989; Hrabetz et al. 2013). Apart from the common mechanism of AChE inhibition, some OP are suspected to induce toxic non-AChE dependent effects (Costa 2006). Clinical as well as experimental in vivo data have shown effects of OP exposure on lung tissue indicating direct toxic effects and an interaction with the immune system (Hulse et al. 2014; Nambiar et al. 2007; Hrabetz et al. 2013). Due to the complexity of the pulmonary system, investigation of the underlying mechanisms is challenging. For the purpose of gas exchange, breathing mechanics and host defense, more than 50 different cell types are present in the lung that act together in a well-balanced composition (Travaglini et al. 2020). Due to limitations in the number of different cell types that can be cultured together, cell–cell interactions and matrix effects that may arise in vivo during lung damage cannot be studied in in vitro cell culture experiments (Liu et al. 2019). As an intermediate step between in vitro cell culture investigations and in vivo studies, precision-cut lung slices (PCLS) as an ex vivo tool are used. The viable lung tissue represents the whole complexity of the organ including all resident cell types in their natural spatial composition (Liberati et al. 2010) and has the potential to substantially reduce the number of in vivo experiments with regard to the 3R principle (Russel and Burch 1959). Due to advantages in standardized preparation and tissue culture, PCLS are frequently used as versatile tool for the investigation of cytotoxic effects, inflammatory activation or alterations of the redox system (Henjakovic et al. 2008; Sauer et al. 2014; Behrsing et al. 2013; Lauenstein et al. 2014). Conservation of the natural lung architecture alongside with presence of viable airways, surrounded by a functioning smooth muscle layer, enables the analysis of compound induced effects on airway response and, therefore, the investigation of novel treatment strategies for diseases like asthma or COPD (Herbert et al. 2019; Martin et al. 1996; Wohlsen et al. 2003).

Among the most frequently studied OP are parathion (WHO classification extremely hazardous; class 1A), malathion (WHO classification slightly hazardous; class III) and their respective biotransformation products paraoxon and malaoxon. We, therefore, used these four compounds to study their effects on airway response, cytotoxicity, inflammatory cytokine expression, alterations of the redox system and intracellular signaling cascades in PCLS. These investigations shed light on potential targets for future improvements in the treatment of OP poisoned patients.

## Methods

### Chemicals

For Tyrode buffer preparation, 2.68 mmol/L KCl (Carl Roth, Karlsruhe, Germany), 1.05 mmol/L MgCl_2_·6H_2_O (Sigma Aldrich, St. Louis, USA), 0.42 mmol/L NaH_2_PO_4_·2H_2_O (Merck KGaA, Darmstadt, Germany), 137 mmol/L NaCl (Carl Roth), 1.8 mmol/L CaCl_2_·2H_2_O (Carl Roth), 22 mmol/L NaHCO_3_ (Carl Roth) and 5.5 mmol/L glucose monohydrate (Merck KGaA) were dissolved in double-distilled water and pH was adjusted to 7.4 by carbogen gassing. PCLS were cultured in Dulbecco’s Modified Eagle's Medium/Nutrient mixture F12 (1:1) without phenol red and l-glutamine (DMEM/F-12; Sigma-Aldrich) supplemented with 1% Penicillin/Streptomycin (Sigma-Aldrich) and 0.1% Gentamycin (Thermo Fisher, Waltham, USA). The low melting point agarose was purchased from Sigma-Aldrich. The OP parathion, paraoxon, malathion and malaoxon were purchased from LGC standards (London, United Kingdom) and stock-solutions (0.1 mol/L) were prepared in acetonitrile (Merck KGaA). For bronchoconstriction experiments, a stock solution (0.1 mol/L in DMEM/F-12) of ACh (Sigma Aldrich) was prepared and stored at − 80 °C. The working solution (50 μmol/L in cell culture medium) was freshly prepared on the day of the experiment. Lysis solution for intracellular protein, cytokine and heme oxygenase 1 (HO-1) detection was prepared using phosphate-buffered saline (Sigma-Aldrich) supplemented with 0.1% Triton-X 100 (Sigma-Aldrich) and cOmplete™ EDTA-free protease inhibitor (Roche, Basel, Switzerland).

### Animals

Male Wistar rats were purchased from Charles River Laboratories (Sulzfeld, Germany) and kept in a standard animal housing unit providing an automated 12 h light/dark cycle and air condition, as described in Herbert et al. (2017). Animals were fed with a standard diet and drinking water ad libitum. Upon arrival in the animal housing, rats were kept for at least seven days before using them for PCLS preparation to allow a proper acclimatization (final weight 300–500 g). All experiments were in accordance with the German Animal Welfare Act of 18th May 2006 (BGB1, I S. 1206, 1313) and the European Parliament and Council Directive of 22nd September 2010 (2010/63/EU).

### PCLS preparation

For preparation of PCLS, a procedure as described previously (Herbert et al. 2017) with some modifications was applied. Briefly, rats were anesthetized by intraperitoneal injection of 75 mg/kg ketamine (Ketavet 100 mg/mL, zoetis Deutschland GmbH, Berlin, Germany) and 10 mg/kg xylazine (Xylasel 20 mg/mL; Selectavet Dr. Otto Fischer GmbH, Weyarn-Holzolling, Germany) and sacrificed by exsanguination. Low melting point agarose (1.5% in DMEM/F-12) was gently heated until boiling and cooled to 37 °C. Lungs were filled with agarose solution until the lung lobules were entirely enfolded. Afterwards, the lung was removed from the thoracic cavity and cooled on ice for 10 min, followed by additional storage for 20 min in 4 °C pre-cooled DMEM/F-12 to allow agarose solidification. Subsequently, tissue cylinders with a diameter of 8 mm were generated using a biopsy punch. The cylinders were sliced into 250–300 µm thick PCLS using a Krumdieck Tissue Slicer (Alabama Research and Development, Munford, USA) with ice-cold Tyrode buffer (pH 7.4) as slicing medium. Slicing medium was changed after five cores and PCLS were collected in pre-cooled DMEM/F-12 until the slicing procedure was finished for all slices. Afterwards, all PCLS were placed in an incubator (HeraCell 240i; Thermo Fisher Scientific) at 37 °C and 5% CO_2_ on a shaker to enable washout of cellular debris and agarose from large airways. The cell culture medium was exchanged every 30 min for 1.5 h and afterwards every 60 min for another 2 h. PCLS were held at 37 °C and 5% CO_2_ until experimental use at the next day.

### Evaluation of bronchoconstriction

PCLS were removed from the incubator and weighted with steel wires in a 24-well plate to prevent floating and were then transferred to the microplate-holder of an inverted microscope (Axio Observer D1, Carl Zeiss AG, Oberkochen, Germany) with an AxioCam HSm camera (Carl Zeiss AG, Germany). Using the AxioVision software (Version 4.8.2.0, Carl Zeiss AG), airway cross sections were observed for signs of vitality such as beating cilia and spontaneous muscle constrictions. At first, the initial airway area was assessed. Afterwards either the solvent control acetonitrile (1%) or the OP (0.001–100 µmol/L) was added to the slices. After 3 min of incubation time of the OP, ACh (0.5 µmol/L final concentration) was added directly into the culture medium. 2 min after ACh application, a second picture was taken to evaluate time-dependent airway constriction. After 60 min, airway relaxation was calculated using the AxioVision software. Relaxation efficacy of solvent exposed slices was set as 100% and response of all OP-exposed PCLS was related to that value.

### Exposure of PCLS with OP compounds

For exposure of PCLS with the OP parathion, paraoxon, malathion or malaoxon, dilutions in DMEM/F-12 were prepared freshly before each experiment, obtaining the final concentrations for exposure between 100 and 2000 µmol/L. Acetonitrile (< 1% for relaxation experiments and < 2% in all other experiments) was used as solvent control and was chosen as it shows low effects on AChE inhibition in human erythrocyte membranes (IC50 of 2.8%) compared to other frequently used solvents such as DMSO (IC50 of 1.1%). In addition, current unpublished work points towards a lower cytotoxicity of acetonitrile in comparison to DMSO, ethanol or methanol in PCLS. Culture medium was replaced by OP-containing medium and PCLS were incubated for 8 or 24 h under standard cell culture conditions (37 °C; 5% CO_2_).

### Analysis of PCLS viability and cell death

To detect effects of OP on PCLS viability, an Alamar Blue assay (Invitrogen, Carlsbad, USA) was performed. The Alamar Blue assay is based on the reduction of resazurin to the fluorescent dye resorufin by metabolically active cells and, therefore, serves as marker for cellular viability. After OP exposure (100–2000 µmol/L), PCLS were incubated with Alamar Blue reagent for 2 h at 37 °C, and fluorescence intensity was detected using a plate-reading photometer (Tecan Infinite 220 PRO, Tecan Group Ltd., Mennedorf, Switzerland) at excitation wavelength of 560 nm and emission wavelength of 590 nm. Signal intensity was referred to the respective solvent control acetonitrile (< 2%). To detect effects of *N*-acetylcysteine (NAC) on PCLS viability, lung slices were pre-treated with 5 mmol/L NAC (Sigma-Aldrich) in DMEM/F-12 for 4 h, and afterwards the medium was replaced by the OP-containing exposure medium for 24 h. For determination of cytotoxicity, an LDH (lactate dehydrogenase) assay was performed (Cytotoxicity detection Kit^PLUS^, Roche), detecting LDH activity in the PCLS supernatant that is released from the cells during cell death. After OP exposure, supernatant was transferred into one well of a 96-well plate. Freshly prepared reaction mix, as described in the manufacturer’s instructions, was added to the wells and incubated for 15 min at room temperature. Absorbance was detected at 490 nm and corrected by the reference wavelength of 605 nm. Cytotoxicity was calculated as % of the absorbance induced by the lysis solution (positive control) provided in the assay kit.

### Analysis of protein content by BCA

The amount of protein in the PCLS after OP exposure (100–1500 µmol/L) was assessed as marker for tissue destruction and as reference for cytokine and HO-1 expression in the sample. After exposure, three PCLS per substance and concentration were sonicated on ice in an Eppendorf cup with 350 µL of lysis buffer using a Bandelin Sonopuls Homogenizer (3 × 5 s; 30% amplitude) (Bandelin electronic GmbH & Co. KG, Berlin, Germany). Sonicated samples were centrifuged (15,000×*g*, 20 min, 4 °C) and supernatant was transferred into an Eppendorf tube. For protein detection, an Uptima BC Assay kit (Interchim, Montluçon, France) was applied according to the manufacturer’s instructions. Absorbance was detected at 562 nm using a plate-reading photometer (Tecan Infinite 220 PRO, Tecan Group Ltd.). The protein concentration was then related to a non-OP exposed, solvent control.

### Cytokine expression evaluated by bioplex assay

To evaluate immunomodulatory effects of the OP (100–1800 µmol/L) on PCLS, cytokines released into the PCLS supernatant and intracellular concentrations were detected using a Bio-Plex Pro™ Rat Cytokine 23-Plex Assay, analyzed on a BioPlex 200 system (Bio-Rad Laboratories, Hercules, USA). Lipopolysaccharide (LPS, 100 ng/mL in DMEM/F12) served as positive control. Intracellular protein was extracted as described in “Analysis of protein content by BCA”. The PCLS supernatant was removed and supplemented with protease inhibitor cocktail solution. All samples were snap-frozen in liquid nitrogen and stored at − 80 °C until further use in the bioplex system. The bioplex assay was performed according to the manufacturer’s protocol with washing steps performed on a HydroFlex microplate washer (Tecan Group Ltd.). Cytokine concentrations of each sample were calculated using the provided assay standards. After normalization to the protein concentration, total amount of cytokines in the PCLS supernatant and cytosol was combined to receive the overall cytokine expression per mg of tissue protein. Cytokine expression is shown as % of the solvent control acetonitrile.

### Glutathione detection assay

For the detection of the reduced (GSH) and oxidized (GSSG) form of glutathione in PCLS, a GSH/GSSG-Glo™ Assay (Promega, Madison, USA) was used as described in the manufacturer’s instructions with slight modifications. After OP exposure (100–1500 µmol/L) each PCLS was transferred into one well of a white 96-well plate (Thermo Fisher Scientific). 50 µL of lysis reagent (total GSH lysis reagent or oxidized GSH lysis reagent) was added directly to the PCLS and incubated for 5 min at room temperature on a plate shaker (850 rpm) to allow tissue lysis. 50 µL of Luciferin Generation solution was added to each well and incubated for 30 min at room temperature in the dark. Afterwards, 100 µL Luciferin Detection Reagent was added and incubated for 15 min. Luminescence was detected using a plate-reading photometer (Tecan Infinite 220 PRO, Tecan Group Ltd.). GSH and GSSG concentrations are depicted as % of solvent control acetonitrile.

#### Glutathione-s-transferase (GST) activity assay

To detect effects of OP exposure (100–1500 µmol/L) on the GST activity, a colorimetric GST activity assay Kit (abcam, Cambridge, UK) was used. After OP exposure, three PCLS per substance and concentration were combined and sonicated (3 × 5 s 30% amplitude) in GST Assay Buffer. After centrifugation (10,000×*g*, 15 min, 4 °C), the intracellular fraction was stored at − 80 °C until further use. GST activity was analyzed as indicated in the manufacturer’s instructions. Absorbance increase between 2 and 10 min was used to obtain GST activity that was afterwards referred to the solvent control acetonitrile.

### Superoxide dismutase (SOD) activity assay

To detect effects of OP exposure on SOD activity in PCLS, a colorimetric Superoxide Dismutase Activity Assay Kit (abcam) was used. In this assay, superoxide is produced by a xanthine oxidase and metabolized into hydrogen peroxide and O_2_ by SOD. Superoxide anions react with WST-1 to produce a formazan dye with an absorbance maximum at 450 nm. The higher the SOD activity is, the less superoxide is present in the sample and, therefore, formazan production is decreased. After OP exposure (100–1500 µmol/L), three PCLS per condition were combined in ice-cold SOD lysis buffer (100 µmol/L Tris/HCl, pH 7.4 containing 0.5% Triton X-100 (Sigma-Aldrich), 5 mmol/L 2-mercaptoethanol (Sigma-Aldrich) and 0.1 mg/mL phenylmethylsulfonylfluoride (PMSF; Roche). Samples were lysed on ice using a Bandelin Sonopuls Homogenizer (3 × 5 s; 30% amplitude). After centrifugation (15,000×*g*, 10 min, 4 °C) sample supernatant was transferred into a fresh tube and snap frozen in liquid nitrogen. Samples were stored at − 80 °C until further use. SOD activity analysis was performed as described in the manufacturer’s instructions. Thereof, SOD activity was calculated for each sample and related to the solvent control acetonitrile.

#### HO-1 and IL-6 detection

After OP exposure, three PCLS per substance and concentration were combined, intracellular protein fraction (HO-1 and IL-6) and supernatant (IL-6) were prepared as described in “Analysis of protein content by BCA” and stored at − 80 °C until further use. For evaluation of nuclear factor 'kappa-light-chain-enhancer' of activated B-cells (NF-κB) activation in OP-induced inflammatory activation, PCLS were exposed to parathion or the solvent control acetonitrile in the presence or absence of 10 µmol/L NF-κB activation Inhibitor VI, benzoxanthiole compound (abcam) for 8 h. DMSO (0.05%) served as solvent control. HO-1 and IL-6 levels were evaluated using commercially available kits (Rat HO-1/HMOX1/HSP32 ELISA Kit; novus biologicals, Littleton, USA and an ELISA Duo Set; R&D Systems, Minneapolis, USA) and were corrected for the protein content of the sample. Results are shown as % of the solvent control acetonitrile.

### Signaling pathway activation bioplex

To analyze effects of OP exposure on signaling pathways, the intracellular fraction was analyzed using a bioplex system. Therefore, specific beads for phospho-c-Jun (Ser63), phospho-p38 MAPK (T180/Y182) and pospho-STAT3 (Tyr705) were used. After 8 h of exposure to 1000 µmol/L of the four OP, three PCLS per exposure were combined, washed with cell wash buffer, and lysed in tissue lysis buffer supplemented with 2 mol/L PMSF (Roche) and 1 × lysis buffer QG (Bioplex Cell Signaling Assay Kit). Samples were sonicated (3 × 5 s; 30% amplitude), centrifuged (15,000×*g*, 20 min, 4 °C) and snap frozen in liquid nitrogen. Afterwards, samples were stored at − 80 °C until further use. The Bioplex assay was performed as described in the manufacturer’s instructions using 200 µg/mL protein per sample. Afterwards, mean fluorescence intensity (MFI) was analyzed on a bioplex 200 system. MFI of the sample is shown as % of the solvent control acetonitrile.

### Data analysis

Data are presented as mean ± standard error of the mean (SEM). Statistical analyses were performed using GraphPad Prism Version 5.04 (GraphPad Software, San Diego, USA). Differences to controls were determined by two-way ANOVA with Bonferroni multiple comparison test. Effects on airway relaxation and NF-κB inhibition were evaluated by one-way ANOVA with Dunnett’s multiple comparison test. A *p* value below 0.05 was considered statistically significant. *p < 0.05; **p < 0.01; ***p < 0.001.

## Results

### Effects of OP exposure on airway relaxation

**Fig. 1:**
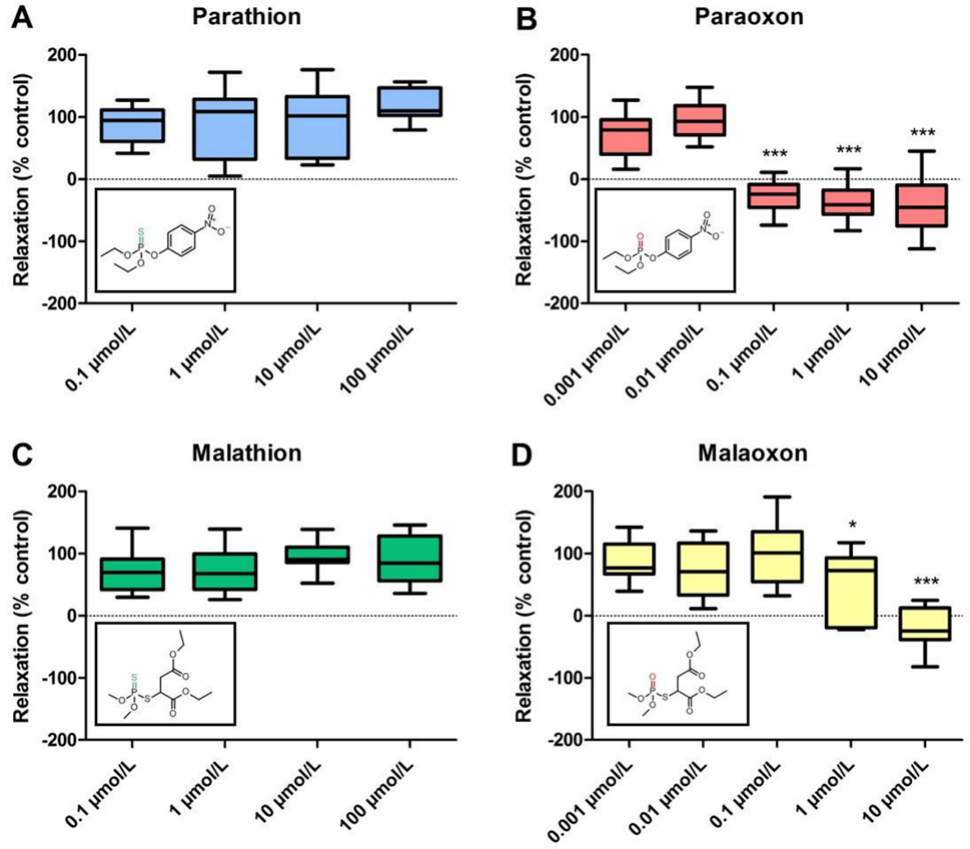
Inhibitory effect of parathion, paraoxon, malathion and malaoxon on airway relaxation after acetylcholine stimulus. PCLS airways exposed for 3 min to parathion (**A**), paraoxon (**B**), malathion (**C**) or malaoxon (**D**) in different concentrations (0.001–100 µmol/L). Upon acetylcholine stimulus (0.5 µmol/L; 2 min), airway constriction was recorded. Subsequent airway relaxation was evaluated after 60 min. Relaxation of the control was set as 100%. Boxplots show mean with minimum and maximum values. Asterisks indicate significant differences to the control (*p < 0.05, ***p < 0.001; *n* = 10 airways from at least three different animals)

For the evaluation of OP effects on airway response, PCLS containing airways were exposed to parathion, paraoxon, malathion or malaoxon and airway relaxation was monitored 60 min after ACh stimulus (0.5 µmol/L). OP exposure without ACh did not result in an alteration of the airway area in the highest applied concentrations (control: 102 ± 3%; parathion: 113 ± 3%; paraoxon: 98 ± 4%; malathion: 104 ± 3%; malaoxon: 109 ± 8%). In addition, maximum constriction of the airway after ACh addition was not significantly different between the four tested OP in the highest applied concentrations (airway area after ACh addition: control: 53 ± 9%; parathion: 51 ± 12%; paraoxon: 45 ± 9%; malathion: 46 ± 10%; malaoxon: 50 ± 8%) and is comparable with other PCLS studies (Wigenstam et al. 2021; Herbert et al. 2017). Exposure with parathion or malathion in concentrations between 0.1 and 100 µmol/L had no significant effect on airway relaxation compared to the solvent control [mean relaxation efficiency 100 µmol/L: parathion (118%, Fig. 1A); malathion (90%, Fig. 1C)]. Airway relaxation after exposure to paraoxon (Fig. 1B) was significantly inhibited by concentrations from 0.1 µmol/L (− 26%) to 10 µmol/L (− 43%). Exposure to malaoxon (Fig. 1D) also led to a reduction in airway relaxation efficiency, that was significant after exposure to 1 µmol/L (53%) and 10 µmol/L (− 20%). Negative values indicate a further increased airway constriction compared to 2 min of ACh incubation as reference value for initial airway constriction.

### Effects of OP exposure on PCLS viability

**Fig. 2:**
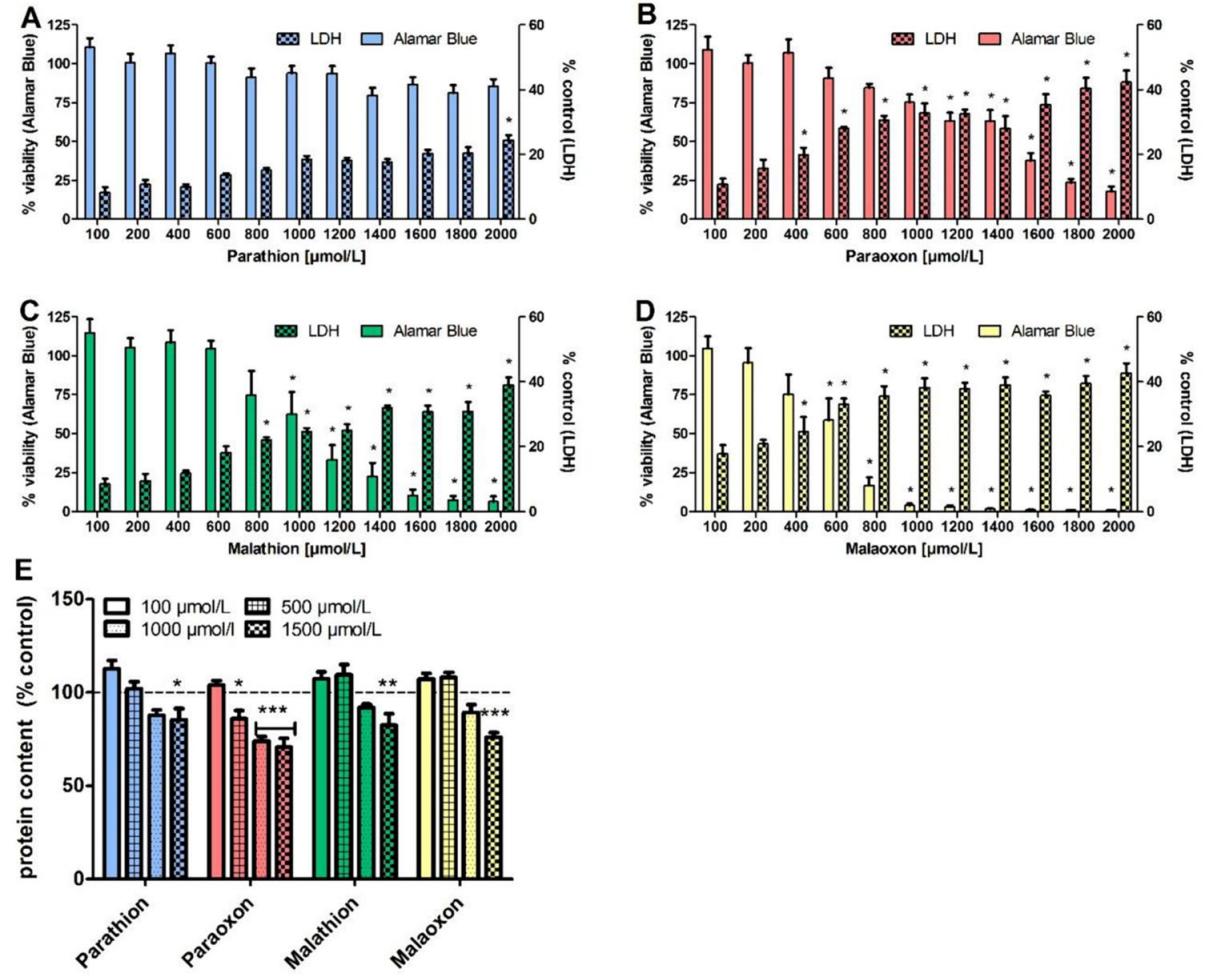
Effects of parathion, paraoxon, malathion and malaoxon on viability, cell death and protein content. PCLS were exposed for 24 h to different concentrations of parathion (**A**), paraoxon (**B**), malathion (**C**) or malaoxon (**D**) (10–2000 µmol/L). Viability was analyzed by Alamar Blue assay and effects on cellular death were evaluated by measurement of LDH release into PCLS supernatant. For detection of severe tissue destruction, intracellular protein content was detected by BCA assay (**E**). Data are presented as % of control (Alamar Blue assay and protein content) or as % of the positive control Triton-X (LDH). Data are shown as mean ± SEM. Asterisk indicate significant differences to control (*p < 0.05; **p < 0.01; ***p < 0.001; *n* = 6 PCLS from three different animals)

To detect effects of OP exposure on the viability of PCLS, intracellular reducing power (Alamar Blue assay), release of LDH and the overall protein content were analyzed after 24 h of exposure with different OP concentrations (100–2000 µmol/L). In addition, we used the Alamar Blue assay and protein detection to exclude possible cytotoxic effects that can influence inflammation and oxidative stress after 8 h of OP exposure. OP exposure for 8 h results in a decrease of viability that was significant only in high concentrations for paraoxon (2000 µmol/L), malaoxon (1600 µmol/L) and malathion (2000 µmol/L) and detection of the corresponding protein content as marker for severe tissue damage indicated no significant differences to the solvent control for all four tested compounds (detailed viability data in Fig S1). After 24 h we made contrasting observations. Using an Alamar Blue assay, a decrease in resazurin reduction was observed for paraoxon, malathion and malaoxon (Fig. 2B–D), while no such effect was detectable after parathion exposure (Fig. 2A). Thereby, significant changes to the solvent control were observed at 1200 µmol/L paraoxon (63 ± 5%), 600 µmol/L malaoxon (59 ± 14%) and 1000 µmol/L malathion (63 ± 14%). Based on the viability, EC_50_ concentrations in a very similar range as the significantly different changes were calculated; i.e. ~ 1400 µmol/L (paraoxon), ~ 600 µmol/L (malaoxon) and ~ 1100 µmol/L (malathion). A complete loss of viability was observed only after exposure to ≥ 1400 µmol/L malaoxon.

An increase in LDH was observed in the PCLS supernatant for several concentrations of paraoxon, malathion and malaoxon, whereas parathion induced a significant effect only in the highest applied concentration. Significant differences to the solvent control were observed at concentrations of 2000 µmol/L for parathion (Fig. 2A); 400 µmol/L for paraoxon (Fig. 2B), 800 µmol/L for malathion (Fig. 2C), and 400 µmol/L for malaoxon (Fig. 2D).

Detection of the protein content of the PCLS after OP exposure and subsequent tissue lysis serves as marker for severe tissue injury. Exposure to the four OP led to a decrease of protein content, that was significant for parathion (85 ± 4%), paraoxon (70 ± 5%), malathion (83 ± 6%) and malaoxon (76 ± 3%) at a concentration of 1500 µmol/L (Fig. 2E).

### Inflammatory activation in PCLS induced by OP

For the detection of inflammatory activation in PCLS after OP exposure, an incubation period of 8 h was used to evaluate inflammatory activation without the bias of severe cytotoxicity and tissue damage that occurs after 24 h of exposure (Fig. 2). LPS (100 ng/mL) as positive control led to a stong expression of pro-inflammatory cytokines after 8 h of exposure (Fig. S2) and pilot tests to find concentration ranges for inflammatory activation revealed OP concentrations of 100, 600, 1200 and 1800 µmol/L as suitable for cytokine detection.

**Fig. 3:**
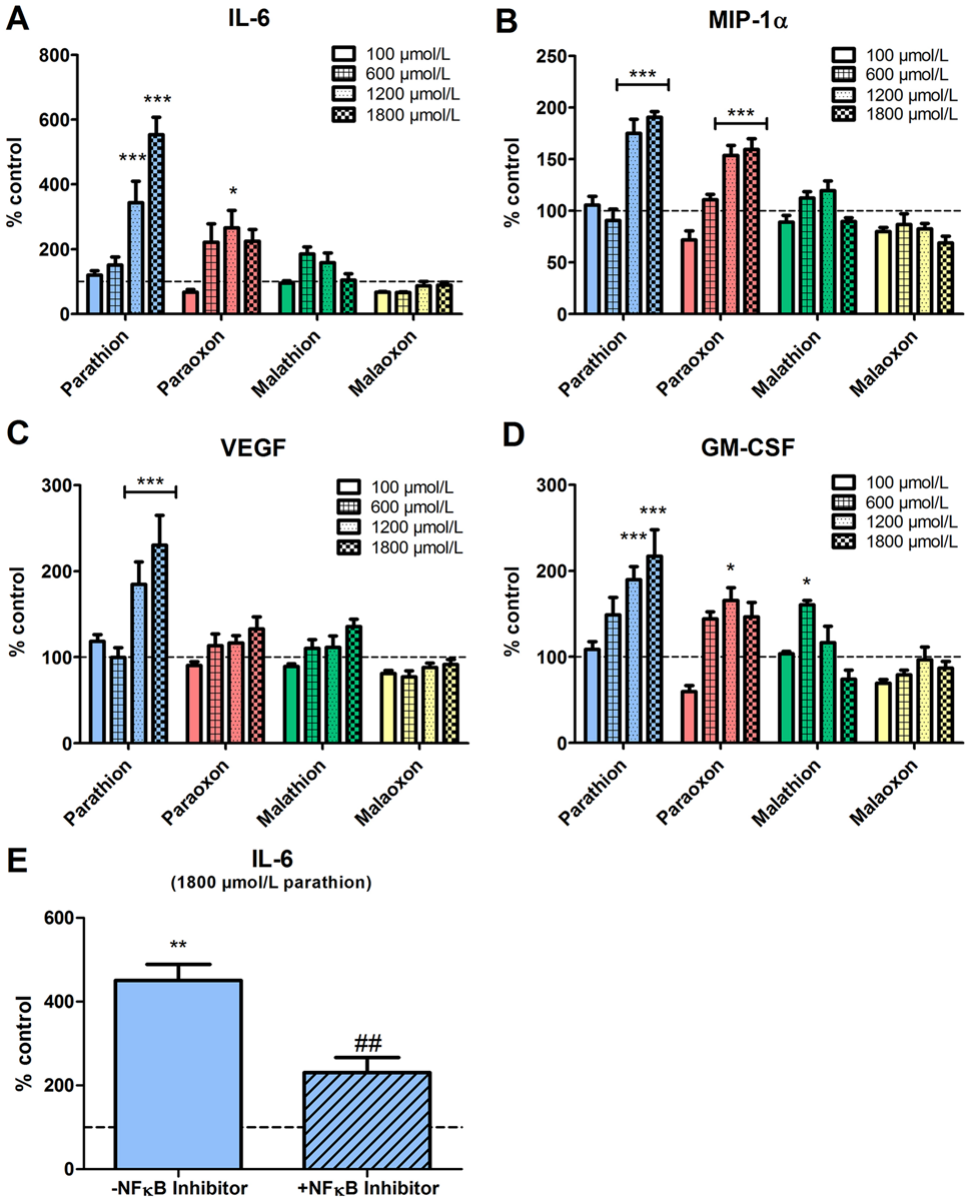
Expression of cytokines in PCLS after parathion, paraoxon, malathion and malaoxon exposure. PCLS were exposed to increasing concentrations of paraoxon, parathion, malaoxon, malathion (100–1800 µmol/L) for 8 h. Cytokine expression of IL-6 (**A**), MIP-1α (**B**), VEGF (**C**) and GM-CSF (**D**) was detected in the supernatant and cytosolic fraction using a Bioplex-system and was afterwards combined to analyze the overall cytokine production. Observed concentrations are corrected for the protein content and are shown as fold increase of control (indicated by dashed line). For evaluation of NF-κB activation, PCLS were exposed to parathion (1800 µmol/L) with or without addition of an NF-κB Activation Inhibitor (10 µmol/L; **E**). Interleukin-6 was detected in the supernatant and cytosolic fraction by ELISA, and afterwards combined to analyze the overall cytokine production. Data are shown as mean ± SEM. Asterisk indicate significant differences to the control (*p < 0.05; **p < 0.01; ***p < 0.001; *n* = 8 samples from four different animals) Hash characters indicate significant differences to parathion exposure without inhibitor (^##^*p* < 0.01; *n* = 8 samples from four different animals)

Induction of IL-6 expression (Fig. 3A) was observed after exposure to parathion (553 ± 53%, *p* < 0.001) and paraoxon (225 ± 35%, *p* < 0.05) at concentrations of 1800 µmol/L and 1200 µmol/L, respectively. In contrast, the expression of vascular endothelial growth factor (VEGF; Fig. 3C) was only increased after parathion exposure (up to 230 ± 35%; 1800 µmol/L, *p* < 0.001), while exposure to the other OP induced no statistically significant differences compared to the control. Expression of granulocyte–macrophage colony-stimulating factor (GM-CSF; Fig. 3D) was moderately upregulated by paraoxon (166 ± 15%; 1200 µmol/L, *p* < 0.05), parathion (217 ± 30%; 1800 µmol/L; *p* < 0.001) and malathion (160 ± 5%; 600 µmol/L, *p* < 0.05) while no changes in GM-CSF expression were observed after exposure to malaoxon. Macrophage inflammatory protein (MIP-1α) expression (Fig. 3B) was significantly increased after exposure to parathion (191 ± 6%; 1800 µmol/L; *p* < 0.001) and paraoxon (160 ± 10%; 1800 µmol/L; *p* < 0.001).

### Cytokine expression after NFκB inhibition

To analyze NFκB signaling pathway activation in OP-induced inflammation, parathion exposed PCLS (1800 µmol/L) were co-incubated with an NFκB inhibitor (10 µmol/L) and expression of IL-6 was analyzed. Cytokine expression was significantly increased to 451 ± 39% (*p* < 0.01 vs. solvent control; Fig. 3E) after parathion exposure. Co-incubation with the NFκB activation inhibitor resulted in a significant reduction of IL-6 expression to 231 ± 36% (*p* < 0.01 vs. parathion exposed PCLS without inhibitor).

### Effects of OP exposure on oxidative stress response

**Fig. 4:**
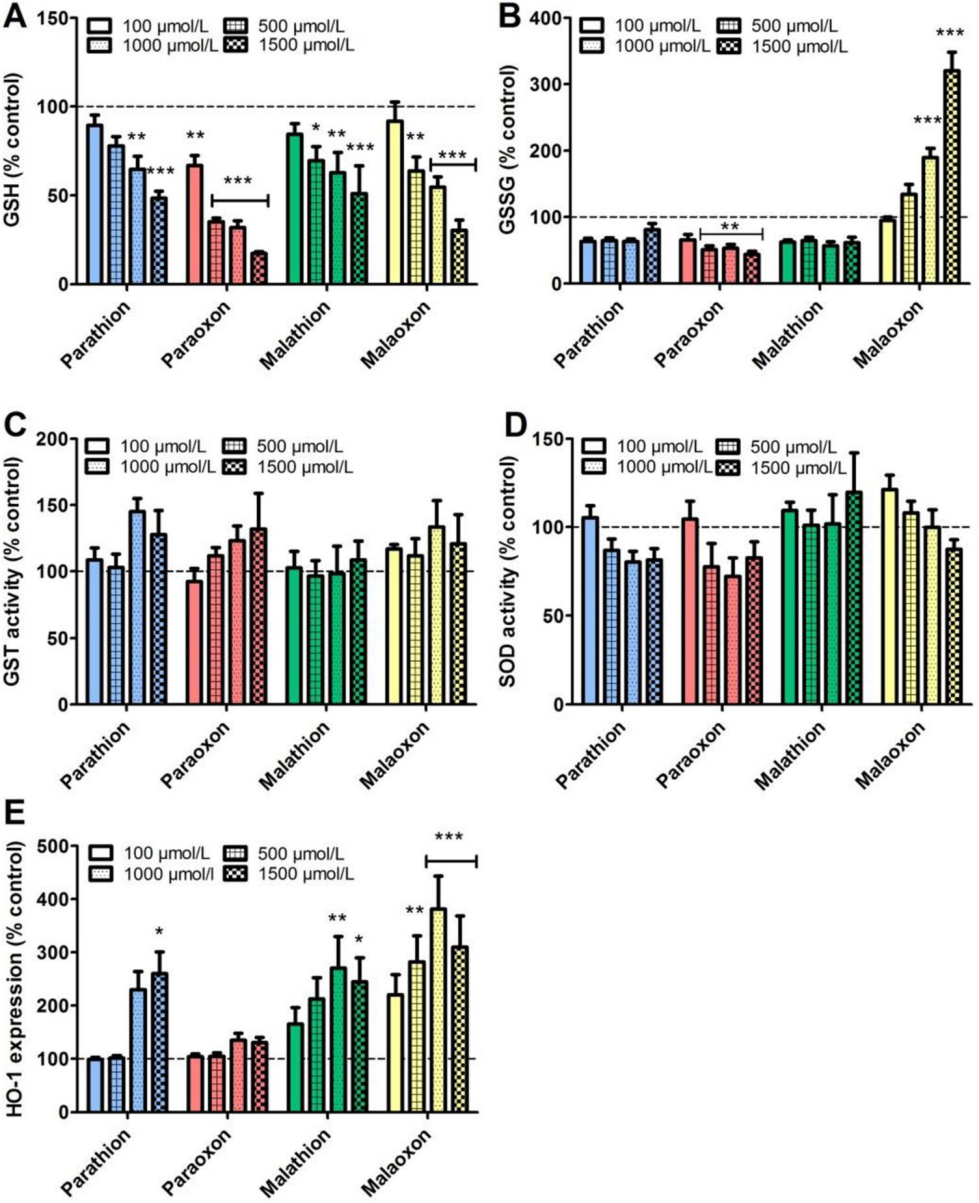
Effect of parathion, paraoxon, malathion and malaoxon exposure on reduced and oxidized glutathione content, GST- and SOD activity and expression of HO-1. PCLS were exposed for 8 h to different concentrations of paraoxon, parathion, malaoxon, malathion (100–1500 µmol/L). Afterwards the intracellular level of reduced (**A**) and oxidized (**B**) glutathione was detected. Furthermore, intracellular activity of GST (**C**) and SOD (**D**) was evaluated. Expression of intracellular HO-1 was evaluated by ELISA and corrected for the protein concentration. All results are shown as % of control (indicated by dashed line). Data are shown as mean ± SEM. Asterisk indicate significant differences to the control (*< 0.05; **p < 0.01; ***p < 0.001; *n* = 6 PCLS from three different animals (GST and SOD Activity Assay) *n* = 8 PCLS from four different animals (GSH, GSSG, HO-1))

To detect effects of OP exposure on the cellular oxidative stress response, the levels of reduced glutathione (GSH) and oxidized glutathione (GSSG) were detected after 8 h of exposure. A decrease of intracellular GSH was observed after exposure to all four OP, resulting in a reduction to 17 ± 1% (paraoxon); 49 ± 4% (parathion); 30 ± 6% (malaoxon) and 51 ± 16% (malathion) at the highest concentration of 1500 µmol/L (Fig. 4A). Detecting intracellular GSSG levels, a significant reduction after exposure to 1500 µmol/L of paraoxon (44 ± 5%) was observed while effects of parathion (81 ± 9%), and malathion (62 ± 8%) were not significant compared to the solvent control. In contrast, a dose-dependent, significant increase of GSSG after exposure to 1500 µmol/L malaoxon (320 ± 28%; *p* < 0.001) was detected (Fig. 4B). To evaluate whether the applied OPs influence the GST activity, which may affect intracellular GSH concentrations, a GST activity assay was performed. After 8 h of exposure, no statistically significant differences to the solvent control were found (Fig. 4C). Exposure to 1500 µmol/L of parathion, paraoxon, malathion and malaoxon resulted in an activity of 127 ± 18%; 132 ± 27%; 108 ± 14% and 120 ± 22%, respectively. As superoxide formation plays a role in the maintenance of the redox system, SOD activity after 8 h of OP exposure was analyzed. SOD activity was not affected in PCLS after 8 h of exposure to the four OP at the highest concentration of 1500 µmol/L (Fig. 4D). For the detection of cellular reactions in response to a possible alteration of the redox system, expression of HO-1 was detected after 8 h of OP exposure (Fig. 4E). While exposure to paraoxon had no effect on HO-1 expression, exposure to 1500 µmol/L parathion resulted in a significantly increased expression (261 ± 40%). A statistically significant induction of HO-1 expression was observed after exposure to 1000 µmol/L of malathion (270 ± 59%; *p* < 0.01), while exposure to malaoxon led to a maximal induction to 310 ± 59% at 1500 µmol/L (*p* < 0.001).

### Pre-treatment with NAC

**Fig. 5:**
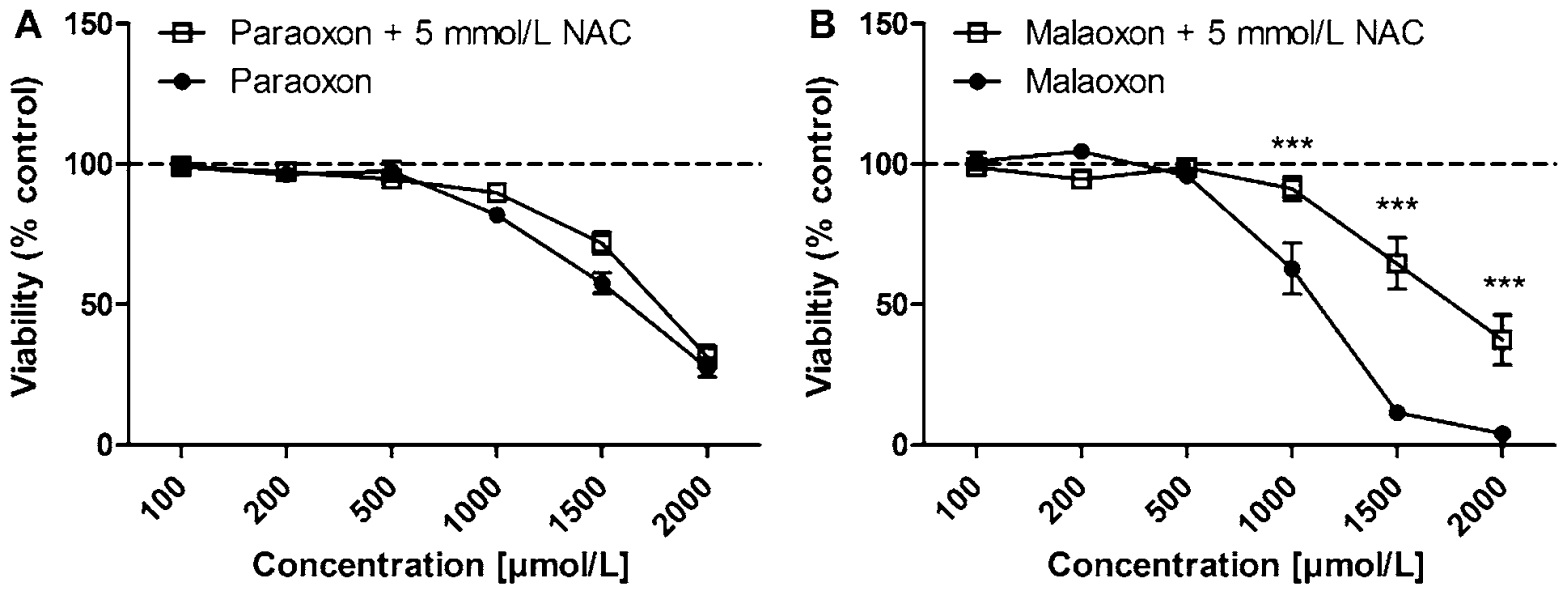
Effects of *N*-acetylcysteine (NAC) pre-treatment on PCLS viability. PCLS were pre-treated with 5 mmol/L of NAC for 4 h. Afterwards medium was replaced by paraoxon (**A**) or malaoxon (**B**) containing medium for 24 h and viability was analyzed by Alamar Blue assay. Viability is shown as % of control (indicated by dashed line). Data are shown as mean ± SEM. Asterisk indicate significant differences to PCLS without NAC pre-treatment (*p < 0.05; *n* = 6 PCLS from three different animals)

While no statistically significant effect of NAC pre-treatment on paraoxon induced cytotoxicity was observed, NAC pre-treatment significantly (*p* < 0.05) reduced the effects of malaoxon on PCLS viability at concentrations of 1000 µmol/L [63 ± 9% (− NAC) vs. 91 ± 4% (+ NAC)]; 1500 µmol/L [11 ± 2% (− NAC) vs. 65 ± 9% (+ NAC)] and 2000 µmol/L [4 ± 1% (− NAC) vs. 37 ± 9% (+ NAC)] (Fig. 5).

### Signaling pathway activation after OP exposure

**Fig. 6:**
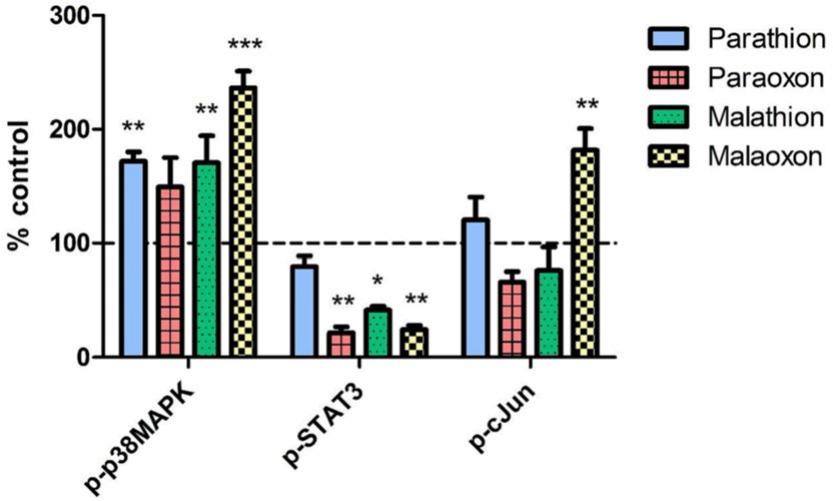
Phosphorylation of signaling pathways after OP exposure. PCLS were exposed to 1000 µmol/L of parathion, paraoxon, malathion or malaoxon for 8 h. Intracellular levels of the phosphorylated proteins p-p38MAPK, p-STAT3 and p-c-Jun were evaluated using a Bioplex system. Data are calculated as % of control (indicated by dashed line) and are shown as mean ± SEM. Asterisks indicate significant differences to the control (*p < 0.05; *n* = 4 samples from four different animals)

To analyze effects of OP exposure on signaling pathways in PCLS, phosphorylation of p38-MAPK, STAT3 and c-Jun was detected using a bioplex assay. An exposure concentration of 1000 µmol/L was used to detect pathway activation without involvement of direct cytotoxic effects. Phosphorylated p38MAPK was significantly (*p* < 0.01) increased by parathion, malathion and malaoxon, which induced the strongest activation (236 ± 15%). In contrast, phosphorylation of STAT3 was significantly (*p* < 0.05) reduced by paraoxon (22 ± 5%), malathion (42 ± 4%) and malaoxon (24 ± 4%) while no significant reduction was observed after parathion exposure (80 ± 9%). Phosphorylation of c-Jun was significantly induced only by malaoxon (182 ± 19%; *p* < 0.01), while exposure to the other compounds did not induce a significant alteration of phosphorylation status (Fig. 6).

## Discussion

Respiratory complications occur in many cases of severe organophosphate poisoning and are a leading cause of death after suicidal or accidental intoxication (Tsao et al. 1990; Hulse et al. 2014). There is increasing evidence that mechanisms other than AChE inhibition may contribute to the high toxicity of OP (Eyer et al. 2010; Xie et al. 2000), e.g. the formation of lung edema, tissue destruction and alterations in the immune response leading to an acute respiratory distress syndrome (Perkins et al. 2011; Lotti 2001). Therefore, toxic effects of parathion, malathion and their respective biotransformation products paraoxon and malaoxon, as representative OP, were investigated in rat PCLS.

### Effects of OP exposure on airway reactivity

As inhibition of the AChE is generally seen as primary toxic mechanism of OP, the AChE inhibitory potential of the four investigated compounds in rat PCLS was assessed. PCLS contain viable airways surrounded by a smooth muscle layer, thus constriction of the airways can be provoked by stimulation with neurotransmitters like ACh (Wohlsen et al. 2003). The constriction is spontaneously reversible due to lung resident AChE activity, which results in relaxation of the airways. Airway response upon OP exposure has previously been studied to find novel treatment strategies in OP poisoning (Herbert et al. 2017, 2019; Wigenstam et al. 2021). Addition of the OP without ACh stimulation failed to induce airway constriction, most probably due to limited nervous stimulation in the PCLS. This was also observed in VX-exposed PCLS (Herbert et al. 2017; Wigenstam et al. 2021). We did not observe significant differences in maximum constriction of the airways between the four applied OP. However, in a PCLS study from Wigenstam et al. (2021), using a slightly different exposure scenario (ACh-OP-ACh instead of OP-ACh), nerve agent exposure in combination with ACh led to an increased bronchoconstriction in PCLS compared to ACh alone. The parent OP parathion and malathion failed to induce an inhibitory effect on airway relaxation, indicating that AChE activity is not impaired (Fig. 1A, C). In contrast, paraoxon and malaoxon significantly decreased airway relaxation in concentrations of 0.1 and 1 µmol/L, respectively (Fig. 1B, D). These results underline the requirement of metabolic activation of thion-OP for substantial AChE inhibition (Eyer et al. 2003; Buratti and Testai 2005). Paraoxon shows a higher inhibitory potential than malaoxon, which is in line with inhibition of rat erythrocyte membrane AChE (second order inhibition rate constant; 2.72 × 10^6^ M^−1^ min^−1^ for paraoxon vs. 4.76 × 10^5^ M^−1^ min^−1^ for malaoxon). In addition, the inhibitory potential is well comparable to human erythrocyte membrane AChE (second order inhibition rate constant; 3.16 × 10^6^ M^−1^ min^−1^ for paraoxon vs. 4.74 × 10^5^ M^−1^ min^−1^ for malaoxon) pointing towards comparable inter-species inhibition data (unpublished data J. Tigges and F. Worek et al. (2020)). The current study nicely shows that AChE-mediated airway response in PCLS is a suitable tool to analyze dose-dependent effects of OP.

### Effects of OP exposure on PCLS viability

PCLS are frequently used to analyze cytotoxic effects to the lung without restriction to particular lung cell types (Neuhaus et al. 2018; Sauer et al. 2014). This is of special interest as it has been shown that OP cytotoxicity is dependent on the particular kind of lung cells used (Angelini et al. 2015). After exposure to OP, a decrease in viability, as well as an increase in LDH release was observed. Only parathion did not induce significant cytotoxic effects (Fig. 2A vs 2B-D). Thereby, decrease in viability and increase in LDH showed a good correlation for the investigated structurally different OP parathion, paraoxon, malathion and malaoxon. Although cytotoxicity of both oxon forms is stronger than that of the related thion-OP, malathion exposure led to a significant decrease in viability compared to the solvent control and an increase in LDH release (Fig. 2C). These findings are in line with observations in human neuronal cells (Bharate et al. 2010; Wang et al. 2019) and pulmonary cells (Angelini et al. 2015). As malaoxon has a much higher AChE inhibiting potency than malathion, a mode of action for cell injury which is different from AChE inhibition is highly likely. The cytotoxic potential is underlined by significantly decreased protein concentrations (Fig. 2E), indicating severe tissue injury (Sauer et al. 2014; Behrsing et al. 2013).

Cytotoxicity is induced at much higher concentrations (up to 5000 fold) than those inducing inhibition of airway relaxation. Therefore, these effects may occur mainly when the lung is directly exposed towards OP, for example after inhalation of nebulized pesticides or by aspiration of OP after suicidal ingestion. If the lung is exposed towards OP exclusively via the bloodstream, effects of AChE inhibition may be lethal before local toxic effects can develop. The parent compound malathion induced dose-dependent cytotoxicity while it failed to provoke a decrease in airway relaxation. Furthermore, malaoxon was more cytotoxic than paraoxon, although paraoxon showed stronger AChE inhibitory potential (effects on different toxicological endpoints summarized in Table 1). This further points towards a different mechanism than AChE inhibition, which is extensively discussed in the literature (Costa 2006; Eyer et al. 2010). It has been found that OP have the potential to bind to more than 50 different proteins in the mouse brain, possibly altering protein function, which may explain AChE independent effects. Interestingly, a particular OP binds only to specific proteins, which might be an indicator for different toxicological modes of action and characteristic effects of particular OP (Lockridge et al. 2005).

**Table 1:** Overview of organophosphate induced effects in PCLS

	Parathion	Paraoxon	Malathion	Malaoxon
Airway relaxation	–	++	–	+
Cytotoxicity	–	+	+	++
Inflammation	++	+	–	–
ROS	+	+	+	++

### Effects of OP exposure on inflammatory activation

Epidemiological studies showed an increased rate of infections in the upper respiratory tract of workers chronically exposed to OP compared to a healthy control group (Hermanowicz and Kossman 1984) and animal studies have shown immunomodulatory effects after exposure to parathion or malathion (Liu et al. 2006; Proskocil et al. 2013; Abdo et al. 2021). PCLS are frequently used as model system for the investigation of substance-induced inflammatory activation indicated by an increased expression of inflammatory cytokines (Henjakovic et al. 2008). We, therefore, analyzed the cytokine expression in rat PCLS after OP exposure. Our results indicate that the cytokine expression in PCLS is highly dependent on the OP. While exposure to parathion and paraoxon caused an increase of the cytokines IL-6, GM-CSF, VEGF and MIP-1α, malathion and malaoxon failed to induce such strong alterations in inflammatory response. Increased expression of these cytokines indicates induction of pulmonary inflammation, macrophage activation and acute lung injury (Fernando et al. 2014; Shibata et al. 2001; Voelkel et al. 2006). As the upregulated cytokines are expressed by activated macrophages, it can be assumed that macrophages play an important role in OP-induced inflammation in PCLS. These suggestions are in line with data obtained from in vitro exposure of macrophage cell lines (Proskocil et al. 2019). Importantly, PCLS contain a mixture of different cells, of which only a portion is involved in immunological response, allowing a more realistic evaluation of inflammatory activation, which is an advantage over single cell-type macrophage cultures. As PCLS were prepared from rat lungs, inter-individual differences need to be considered. In the present study, we observed different basal immunological activation between animals, which necessitates normalization to the individual controls which is a well-known limitation in PCLS (Sauer et al. 2014).

### NfĸB-signal transduction in OP poisoning

Expression of the above mentioned cytokines is dependent on activation of the NF-κB signal transduction pathway (Kiriakidis et al. 2003; Liu et al. 2017). In brief, activation of cytokine receptors, pattern recognition receptors or T- and B- cell receptors results in stimulation of the IκB kinase (IKK) complex, leading to phosphorylation and subsequent degradation of IκBα. Thereafter, the NF-κB dimers p50/RelA and p50/c-Rel translocate to the nucleus, acting as transcription factor for the induction of target genes at the κB response element (Liu et al. 2017). To evaluate whether inflammatory activation induced by OP is NF-κB dependent, the effects of an IKK-β inhibitor on IL-6 expression after parathion exposure were evaluated (Fig. 3E), as this combination has provoked the strongest response in the Bioplex screening (Fig. 3A). IL-6 expression was significantly decreased after co-incubation of parathion with the IKK-β inhibitor, which demonstrates an activation of the NF-κB signal transduction pathway in response to parathion exposure. It has been found that the NF-κB signal transduction pathway is activated by parathion in differentiated human macrophages and that parent compounds show a stronger activation potential than their metabolites, which is in line with our observations of cytokine expression (Proskocil et al. 2019). The successful inhibition of inflammatory activation confirms PCLS as a potential model system to study the use of candidate therapeutics for treatment of pulmonary inflammation after OP exposure and may be a potential explanation for alterations of the immune system observed in OP-poisoned patients. Anti-inflammatory drugs (COX2 inhibitors) have been already used in an in vivo OP model (rat) (Chapman et al. 2019) and could be evaluated in PCLS.

### Effects of OP exposure on antioxidative defense

Elevated levels of reactive oxygen species (ROS) like superoxide or hydrogen peroxide can cause substantial damage to cellular organelles, lipids, proteins and DNA which may result in apoptosis (Sies et al. 2017). Reduced GSH acts as intracellular antioxidant that is used by the glutathione peroxidase for detoxification of ROS. The oxidized GSSG is afterwards reduced to GSH by a glutathione reductase (Rahman et al. 2005). Antioxidative enzymes like SOD have a key role in antioxidant defense by eliminating intracellular superoxide (Afonso et al. 2007). In addition, cells that are under oxidative stress respond with the expression of antioxidative proteins like HO-1, which has tissue protective properties by oxidative cleavage of free heme groups (Ryter and Choi 2005).

Alterations of the antioxidative system were observed in patients after exposure to OP suggesting a role of ROS in OP poisoning (Banerjee et al. 1999; Seth et al. 2001). In PCLS, intracellular GSH levels can be used as marker for the induction of oxidative stress (Sauer et al. 2014). In the present study a significant decrease in intracellular GSH levels for all applied OP in concentrations below those inducing cytotoxic effects was observed (Fig. 4A). Reduced levels of intracellular GSH indicate a cellular reaction towards ROS in response to oxidative stress. Interestingly, the oxidized GSSG was only significantly upregulated after malaoxon exposure, indicating a stronger impairment of oxidative defense of malaoxon than the other OP (Fig. 4B). It has also been shown that loss of intracellular GSH is a mechanism that regulates redox signaling and cell death which may be an explanation for decreased GSH levels without an increase of GSSG (Franco and Cidlowski 2012). As decrease of GSH can be related to an increased biotransformation of OP by the glutathione-*S*-transferase (GST) (Fujioka and Casida 2007), GST activity in PCLS after OP exposure was analyzed. No significant changes in GST activity were observed, indicating that reduced GSH levels are not related to an increase in metabolic activity (Fig. 4C). Impairment of SOD activity seems to be not involved in OP-induced alterations of the antioxidative defense system (Fig. 4D). Expression of HO-1, indicating cellular response to oxidative stress, was significantly upregulated following parathion, malathion and malaoxon exposure, with malaoxon inducing the strongest effects (Fig. 4E). These findings further underline the diversity of OP in induction of antioxidative response and that malaoxon provokes a stronger alteration of the redox system than malathion, parathion or paraoxon. It has been shown that malathion induces oxidative stress in the rat brain that could be due to inhibition of the complex IV of the respiratory chain (Delgado et al. 2006). However, the exact mechanism leading to ROS generation after malaoxon exposure remains elusive.

As medical countermeasure, we analyzed whether pre-incubation of PCLS with the antioxidative GSH precursor NAC has an impact on OP-induced cytotoxicity. Viability after malaoxon but not after paraoxon exposure was significantly improved after pre-incubation with NAC (Fig. 5), indicating that malaoxon induced cytotoxicity is at least in part dependent on alterations of the redox system. Future studies could evaluate the therapeutic efficacy of antioxidative drugs such as vitamin E that were already evaluated in vivo (John et al. 2001).

### Effects of OP exposure on signaling pathway activation

Mitogen activated protein kinase (MAPK) signaling pathways like the c-Jun N-terminal kinase (JNK), p38MAPK or extracellular signal regulated kinase (ERK) play an important role in a variety of cellular processes like cell differentiation, apoptosis and oxidative stress response (Morrison 2012). In our study we evaluated the intracellular levels of phosphorylated p38MAPK, Signal transducer and activator of transcription 3 **(**STAT3) and c-Jun as key players in the p38MAPK, ERK and JNK pathways, respectively. An increase in p38MAPK phosphorylation was observed for all investigated OP and was accompanied by a reduction in phosphorylation of STAT3 (Fig. 6). p38MAPK is activated in response to a variety of different noxious stimuli including UV, heat and cytokine stimulation. Phosphorylation results in transcription of target genes involved in cell cycle control and cell death (Morrison 2012). It has been shown that activation of p38MAPK in combination with a decreased activity of the ERK pathway (e.g. STAT3) is important for the induction of apoptosis (Xia et al. 1995) and that MAPK are involved in the toxicity of several OP (Farkhondeh et al. 2020). The JNK signal transduction pathway is involved in several cellular mechanisms including response towards oxidative stress (Ki et al. 2013; Kamata et al. 2005). Significant changes in c-Jun phosphorylation were only observed after malaoxon exposure, which is in line with the oxidative stress inducing potential of this OP (Figs. 4 vs. 6). The observed changes in activation of the investigated pathways underline the cytotoxic and oxidative stress inducing potential and it can be assumed that MAPK activation is involved in the observed toxic effects of OP in rat PCLS.

## Conclusions

The OP parathion, malathion and their respective biotransformation products paraoxon and malaoxon induce distinct toxicological effects in PCLS. Effects of these OP on airway relaxation are well comparable with their effects on AChE inhibition. Induction of cytotoxic effects is mediated by much higher concentrations than functional impairment via AChE inhibition. Interestingly, OP showed different potency for inflammatory activation as well as for the disturbance of antioxidative response (summarized in Table 1). These results indicate that OP exert compound-specific toxic effects in rat lung tissue beyond AChE inhibition. PCLS are a valuable tool for the investigation of direct toxic effects (e.g. cytotoxicity or inflammatory activation) as well as for the analysis of tissue architecture and physiological response (airway relaxation), which offers unique possibilities to investigate multiple effects of OP exposure in a single tissue culture system. Future studies using PCLS may address the underlying mechanisms of non-AChE related effects by RNA expression, protein analysis and potential effects on other signaling pathways. In addition, the combined use of various precision cut tissue slices (e.g. lung, liver and kidney) might provide insights into effects of metabolic activation and detoxification.

## Supplementary Information

Below is the link to the electronic supplementary material.

Supplementary file1 (DOCX 495 kb)

## Abbreviations

ACh: Acetylcholine
AChE: Acetylcholinesterase
DMEM: Dulbecco’s Modified Eagle's Medium
ERK: Extracellular-signal regulated kinases
GM-CSF: Granulocyte–macrophage colony-stimulating factor
GSH: Glutathione
GSSG: Glutathione disulfide
GST: Glutathione-*S*-transferase
HO-1: Heme oxygenase-1
IC50: Inhibitory concentration 50%
IKK: IĸB kinase
JNK: C-Jun N-terminal kinase
ROS: Reactive oxygen species
IL-6: Interleukin-6
LDH: Lactate-dehydrogenase
LPS: Lipopolysaccharide
MAPK: Mitogen-activated protein kinase
MFI: Mean fluorescence intensity
MIP-1Α: Macrophage inflammatory protein 1A
NAC: *N*-Acetylcysteine
NFĸB: Nuclear factor ‘kappa-light-chain-enhancer’ of activated B-cells
OP: Organophosphorus compound pesticides
PCLS: Precision-cut lung slices
PMSF: Phenylmethylsulfonylfluoride
SEM: Standard error of the mean
SOD: Superoxide dismutase
STAT3: Signal transducers and activators of transcription 3
VEGF: Vascular endothelial growth factor
