# Supplementary material for: Organophosphorus pesticides exhibit compound specific effects in rat precision-cut lung slices (PCLS): mechanisms involved in airway response, cytotoxicity, inflammatory activation and antioxidative defense
Source: Arch Toxicol. 2021 Nov 15;96(1):321–34. doi: 10.1007/s00204-021-03186-x (PMC8748323; doi:10.1007/s00204-021-03186-x)
Supplement: Supplementary file 1 — Supplementary file1 (DOCX 495 kb) [file 204_2021_3186_MOESM1_ESM.docx]

**Supplementary Materials:**


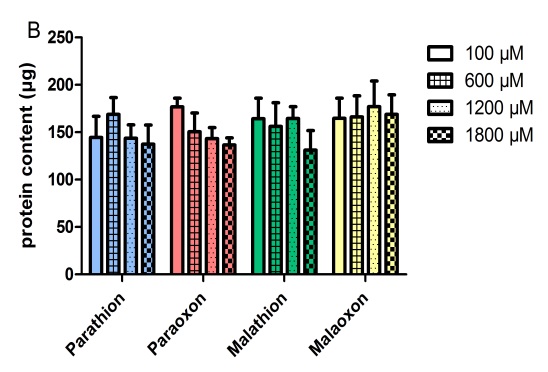

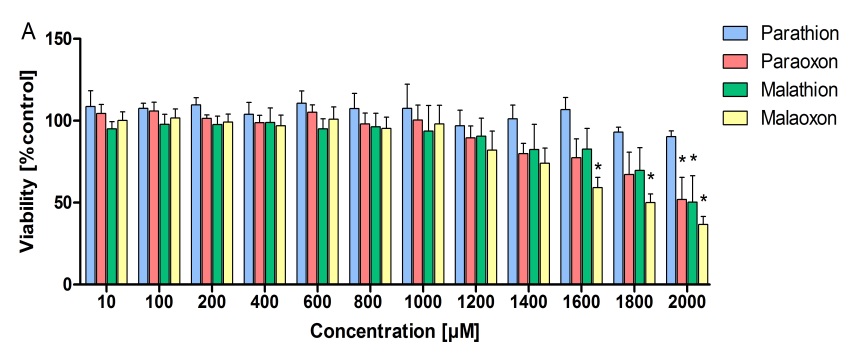


**S1: Viability of PCLS after 8 h OP exposure (A) and corresponding protein content (B).** To determine optimal concentrations of the OP substances for analysis of cytokine release and induction of oxidative stress, PCLS were treated for 8 h with either paraoxon, parathion, malaoxon, malathion (10 – 2000 µmol/L) or the solvent control acetonitrile. Viability was analyzed by Alamar Blue assay and protein content was measured by BCA assay. Results are shown as % of the solvent control acetonitrile (A) or total protein content (B). Data are shown as mean ± SEM. Asterisk indicate significant differences to the solvent control (*p<0.05; n=3).


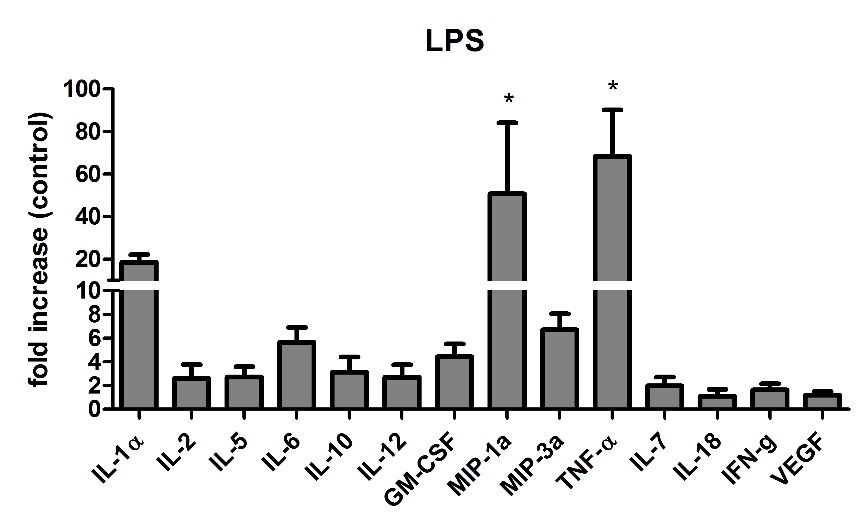


**S2: Effects of the positive control LPS on cytokine expression in PCLS.** To verify that PCLS are suitable for the investigation of cytokine expression, PCLS were exposed for 8 h with 100 ng/ml LPS and cytokine expression was detected by a multiplex assay. Results are shown as % of the untreated control. Data are shown as mean ± SEM. Asterisk indicate significant differences to the solvent control (*p<0.05; n=3).
